# Ultrasound improves the physicochemical and foam properties of whey protein microgel

**DOI:** 10.3389/fnut.2023.1140737

**Published:** 2023-04-11

**Authors:** Zhaoxin Wang, Haibo Zhao, Haiteng Tao, Bin Yu, Bo Cui, Yan Wang

**Affiliations:** ^1^College of Food Science and Engineering, Qilu University of Technology, Shandong Academy of Sciences, Jinan, Shandong, China; ^2^State Key Laboratory of Biobased Material and Green Papermaking, Qilu University of Technology, Shandong Academy of Sciences, Jinan, Shandong, China; ^3^College of Food and Biological Engineering, Qiqihar University, Qiqihar, Heilongjiang, China

**Keywords:** whey protein microgel, ultrasound, physicochemical properties, foam properties, modification

## Abstract

Whey protein microgel (WPM) is an emerging multifunctional protein particle and methods to improve its functional properties are continuously being explored. We developed a method to prepare WPM by heat-induced self-assembly under different ultrasound power (160, 320, 480, and 640 W/cm^2^) and characterized the particle size, surface hydrophobicity, disulfide bond, viscosity, and foam properties of WPM. Ultrasound increased the particle size of WPM-160 W to 31 μm. However, the increase in ultrasound power gradually reduced the average particle size of samples. The intrinsic fluorescence spectrum showed that ultrasound unfolded the structure of whey protein and exposed more hydrophobic groups, which increased the surface hydrophobicity of WPM. In addition, infrared spectroscopy suggested ultrasound decreased the α-helix content of WPM, implying an increase in the flexibility of protein molecules. The disulfide bond of WPM was broken by ultrasound, and the content of the-SH group increased correspondingly. The rheology indicated that the apparent viscosity decreased with the increase of ultrasonic power. Compared with the control, the ultrasonicated WPM displayed higher foam ability. Ultrasound improved the foam stability of WPM-160 W but destroyed the foam stability of other samples. These results suggest that proper ultrasound treatment can improve the physicochemical and foam properties of WPM.

## 1. Introduction

Microgel is swollen polymer particles prepared by cross-linking between biomolecules to retain solvent molecules and thus form a three-dimensional network structure (1). Microgel is valuable for research as it is responsive to temperature and pH and has polymeric and granular properties. Microgel transports sensitive bioactive components such as anthocyanins, growth factors, antibodies, etc. (2). The applications range of microgels has been gradually expanded by different modifications. For example, its application can be extended by improving the microgel emulsification properties through the Maillard reaction (3).

Whey proteins are widely applied in the food industry because of their foaming, gelling, and emulsifying properties. In recent years, research on whey protein microgel (WPM) has gradually increased. WPM has been utilized to stabilize Pickering emulsions (4) and foam (5). Lee and Duggan (6) found that WPM had better foam stability than WPI, but poorer foaming ability due to the increased particle size and viscosity. Many researches improved foam properties by exploring the formation conditions and modification methods of WPM. For example, the WPM was modified by an acetyl grafting reaction to improve its foam ability (7). However, the safety of chemical modification is a concern. The physical modification is worth exploring because of its low cost, non-toxic side effects, and low damage to the nutritional value of the protein.

Ultrasound is a convenient and effective physical method to improve the functional properties of proteins. During the ultrasound process, the cavitation phenomenon is caused by the violent collapse of the microbubbles after increasing to the maximum size. Ultrasound has been widely used to strengthen the foam ability of various proteins, such as whey protein (8) and soybean isolate protein (9). Ultrasound induced protein to expose hydrophobic regions and enhanced its foaming ability (10). WPM is formed by denaturation and cross-linking of WPI during heating, so we speculated that the addition of ultrasound treatment may affect the formation of WPM during the heating process. Morales et al. (9) found that the foam property of soybean protein treated with ultrasound at 85°C was significantly higher than that of samples treated with ultrasound at room temperature. This proved that the combined effect of heating and ultrasound produced a synergistic phenomenon that can improve the physicochemical properties of the proteins.

As far as we know, there are few reports on the application of ultrasound to modify WPM during the heating process. To investigate the effect of ultrasound on WPM formation during the heating process, we applied ultrasound of different power in the preparation process. The prepared WPM was characterized by particle size, Fourier transform infrared spectroscopy, intrinsic fluorescence, and sulfhydryl group. These properties are closely related to the foam properties of protein. We investigated the effect of ultrasound on the functional properties of proteins by evaluating the foam properties and foam structure of WPM. This research can suggest new ideas for modifying WPM and widen the path for further application of WPM in food foam products.

## 2. Materials and methods

### 2.1. Materials

Whey protein isolate (WPI, Hilmar TM9010) was obtained from the Tianjin Milkyway Wei Ye Import and Export Trading Co. Ltd. (Beijing, China). The ingredients of WPI were 87.5% protein, 4.8% water, 2.5% ash, 1.5% fat, and 1.0% lactose. Rhodamine B and 1-anilino-naphthalene-8-sulfonate (ANS) were obtained from Sinopharm Chemical Reagent Co., Ltd. (Shanghai, China). Urea, ethylene diamine tetraacetic acid (EDTA), 5, 5’-Dithiobis-(2-nitrobenzoic acid), and glycine (Gly) were bought from Macklin Biochemical Technology Co., Ltd. (Shanghai, China). Tris (hydroxymethyl) aminomethane (Tris) was purchased from Beijing Coolaber Technology Co., Ltd. (Beijing, China). Other chemicals were of analytical reagent grade.

### 2.2. Preparation of WPM

WPI powder was dissolved in distilled water to form a 3% (*w*/*w*) solution, and 0.02% (*w*/*v*) sodium azide was used as a bacterial inhibitor. The WPI solution was stirred at room temperature (25°C) for 2 h and then left overnight at 4°C to allow complete hydration. The pH of the WPI solution was adjusted to 6.3 by using NaOH and HCl (0.5 mol/L). Subsequently, the 100 mL of WPI solution was heated in a water bath at 85°C for 15 min. At the same time, ultrasound treatment (probe diameter 13 mm, working for 5 s, stop for 5 s) was performed using an ultrasound processor (VCX800, Sonics & Materials, Inc., United States). Ultrasound treatment was selected at 20, 40, 60, and 80% of the maximum power (800 W), which is 160 W, 320 W, 480 W, and 640 W in the manuscript. The WPM was rapidly cooled to room temperature by exposing to cold water. The prepared WPM under different ultrasound power was named WPM-160 W, WPM-320 W, WPM-480 W, and WPM-640 W, and the sample without ultrasound treatment was used as the control.

### 2.3. Particle size

The particle size of WPM was measured by laser diffraction method (Mastersizer 3,000, Malvern, UK). The real and imaginary refractive indices were set at 1.53 and 0.01, respectively. The measurement procedure was carried out at room temperature (25°C).

### 2.4. Zeta potential

The zeta potential of WPM was determined with a zeta potential analyzer (Zetasizer Nano ZSE, Malvern, UK). The sample was diluted 50 times (*w*/*w*) with ultrapure water to avoid multiple scattering effects that could affect the measurement results. The samples were left for 3 min until they were stable. The electrophoretic mobility of the particles was measured and converted into zeta potential values. Each sample was repeated five times.

### 2.5. Intrinsic fluorescence

Intrinsic fluorescence spectroscopy was employed to assess the structural transitions of samples using a fluorescence spectrophotometer (F-4500, Hitachi, Japan). The measurement of intrinsic fluorescence was performed on the basis of the method of Gao et al. (11). The excitation wavelength of 295 nm was set, and the emission spectra ranging from 280 to 400 nm was recorded. The entrance and exit slits were set to 5 nm.

### 2.6. Fourier transform infrared spectroscopy

The FT-IR Nicolet i10 spectrometer (Nicolet i10, Madison, United States) was equipped with an attenuated total reflection (ATR) sampling accessory for recording FTIR spectra of WPM. WPM powder samples were obtained by freeze-drying. The spectra were measured (64 scans) and recorded in the wave number range between 4,000–400 cm^−1^. The measurements were repeated three times for each sample and averaged. Variations in the secondary structure of the protein were obtained by analyzing changes in the WPM amide Ι region (1,700–1,600 cm^−1^) using PeakFit version 4.12 software (SPSS, Inc., Chicago, United States). Curves were fitted by the method of Fevzioglu et al. (12).

### 2.7. Surface hydrophobicity

The surface hydrophobicity of samples was determined using 1-anilino-naphthalene-8-sulfonate (ANS) fluorometric assay with a UV spectrophotometer (UV-6100, Shimadzu Corporation, Japan) (5). Dilute WPM with phosphate buffer (pH 7.2) to five concentrations between 0.005 and 0.025% (*w*/*w*). 20 μl of ANS solution was added to 4 mL of WPM suspension, vortexed, and avoided light reaction for 5 min. The relative fluorescence intensity of each sample was measured at excitation and emission wavelengths of 365 and 484 nm (slit width 5 mm).

### 2.8. Free sulfhydryl content

Ellman’s reagent colorimetry was used to determine the free sulfhydryl content of samples using a UV spectrophotometer (TU-1810, Persee Corporation, China). The measurement method was partially modified according to the previous method (13). First, 5 mL of Tris-Gly buffer at pH 8.0 (0.086 mol/L Tris, 0.09 mol/L Gly, 8 mol/L urea, 4 mmol/L EDTA) was added to 0.5 mL (1 mg/mL) of WPM suspension and then mixed with 20 μL of Ellman’s reagent (4 mg/mL 5,5’-Dithiobis-(2-nitrobenzoic acid), Tris-Gly buffer). Afterwards, the mixture was vortexed and kept in the dark at room temperature for 20 min. After the reaction was completed, the absorbance of the sample was measured at 412 nm.

### 2.9. Apparent viscosity

The apparent viscosity of samples was measured using a rotary rheometer (MCR 302, Anton Paar, Austria). A 50 mm diameter plate was selected, and the temperature was set to 25°C to scan the WPM sample at a shear rate of 0.01–100 s^−1^.

### 2.10. Foam ability and stability

The method of Xiong et al. (10) was partially modified to measure the foam properties. The sample suspensions prepared in Section 2.2 were whipped at 10000 rpm for 3 min using a high-speed disperser (Ministar T25 digital, IKA, German). The foam generated was rapidly transferred to a 100-mL glass measuring cylinder, the volume of foam was observed, and the total height of the foam and the liquid layer was recorded. The foam volume was calculated from the difference between the foam and liquid layer height, and later the foam stability was calculated by measuring the change in foam volume after 10 min of the foaming process.


$$\mathrm{Foam ability}\;\left(\%\right)=\frac{V_{1}}{V_{0}}\times 100$$

$$\mathrm{Foam stability}\;\left(\%\right)=\frac{V_{2}}{V_{1}}\times 100$$

where V_0_ is the initial liquid volume, V_1_ is the original foam volume, and V_2_ is the foam volume after 10 min. All experiments were performed three times, and the mean of the results was recorded.

### 2.11. Micrographs of foams

The different samples were first stained with rhodamine B and then foam was prepared using the method of Section 2.10. The manufactured foam was immediately moved to the microscope and observed with a 50× magnification. The micrographs of foams were recorded at 0, 5, and 10 min using a Zeiss fluorescence microscope (BX53F, Olympus, Japan) to visualize the foam variations during the defoaming process. Image-Pro.2.10 Statistical analysis (version 2.10, Media Cybernetics, United States) was used to document the foam size and the number of micrographs.

### 2.12. Statistical analyses

Each measurement was performed three times, followed by statistical analysis of the means and standard deviations using the SPSS 19.0 software package (version 19, IBM Software, United States). One-way analysis of variance (ANOVA) tests was carried out, and significant differences were defined as having values of *p* < 0.05 using Duncan’s multiple range test.

## 3. Results and discussion

### 3.1. Particle size

**Figure 1:**
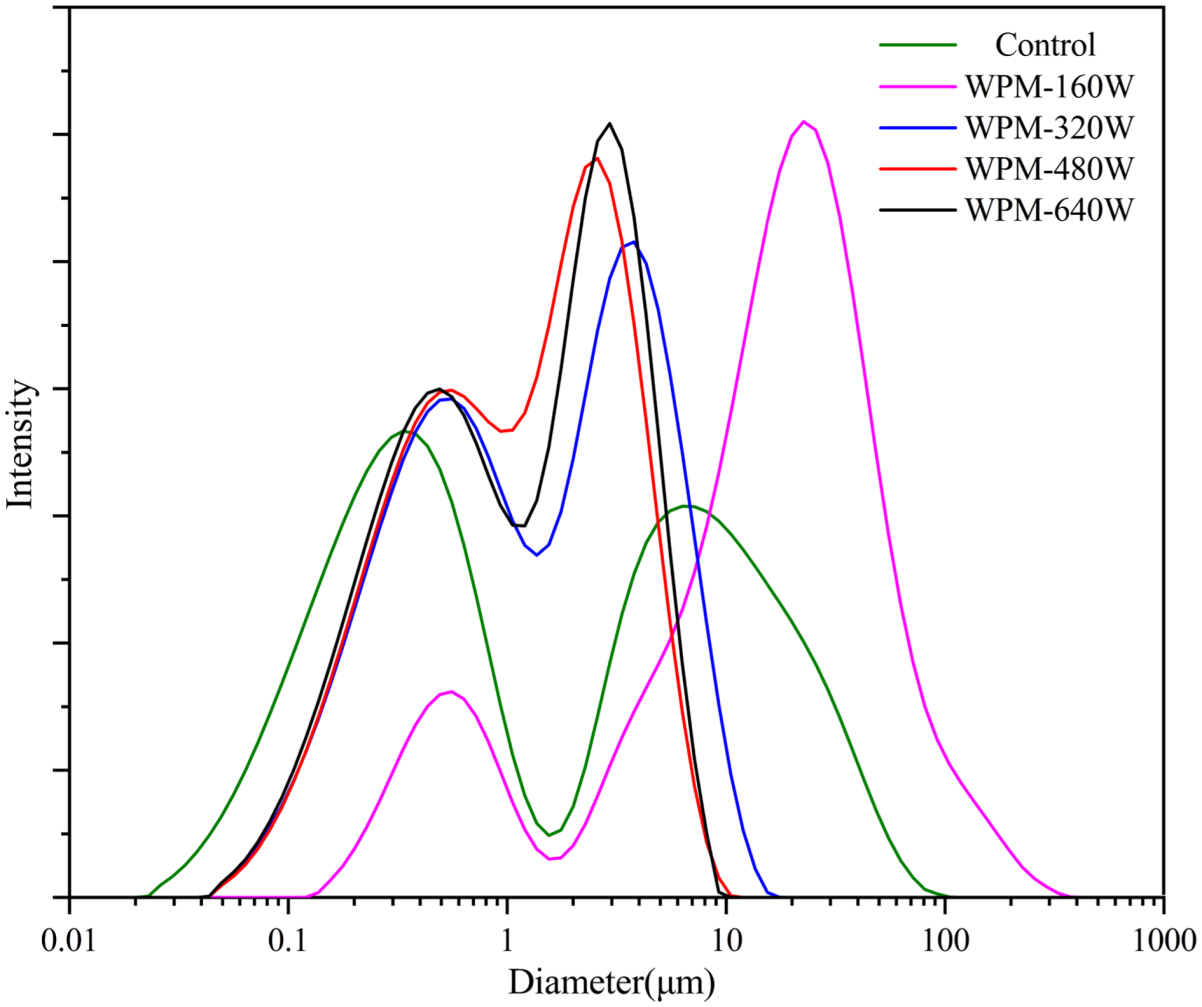
Particle size distribution of WPM prepared under different ultrasound powers.

The particle size and distribution of WPM were evaluated to investigate the effect of ultrasonic power on WPM formation, and the results were shown in Figure 1 and Table 1. The particle size distribution of all WPM showed a bimodal peak. The first peak mainly appeared at 0.1–1 μm, and the second peak primarily appeared in the range of 3–10 μm. The first peak agreed with the particle size range of WPM prepared by other researchers (14). The presence of the second peak, probably owing to the low charge repulsion and non-covalent attraction between WPM molecules, inducing aggregation to form a large particle size of WPM. Gao et al. (11) found a bimodal distribution of sample particle size when ultrasound treatment of WPI was performed at the isoelectric point, which was caused by a reduction in electrostatic repulsive forces. The particle size of WPM-160 W increased by 24 μm and was more widely distributed than the control, which may be due to the aggregation of the WPM fragments by low-power ultrasound. Gülseren et al. (15) found that low-power ultrasound treatment of bovine serum proteins caused aggregation of small fragments and thus increased particle size. When the ultrasound power increased to 480 W/cm^2^, the average particle size of the samples decreased to 2.0 μm, and the distribution range was gradually concentrated. This may be due to the cavitation effect of ultrasound that generated a strong shear force, which resulted in the rupture of large protein aggregates. Gamlath et al. (16) also pointed out that ultrasound broke whey protein particles into smaller ones (< 6 μm), and large aggregates were rarely found. Although the particle size of WPM-640 W was slightly larger than that of WPM-480 W, there was no statistically significant difference. This may be explained by the increased temperature, which reduced the effectiveness of ultrasound (17).

**Table 1:** Zeta-potential, surface hydrophobicity, -SH content, and average particle size of WPM prepared under different ultrasound powers.

Sample	Zeta potential (mV)	Surface hydrophobicity	-SH content (μmol/g)	Particle average size (μm)
Control	−13.8 ± 1.2^d^	2841.7 ± 152.2^e^	9.00 ± 0.1^e^	7.60 ± 0.2^b^
WPM-160 W	−15.4 ± 0.2^c^	3752.3 ± 134.6^d^	21.4 ± 0.0^d^	31.6 ± 0.2^a^
WPM-320 W	−16.4 ± 1.0^bc^	4050.6 ± 220.4^c^	29.8 ± 0.5^c^	2.80 ± 0.1^c^
WPM-480 W	−17.2 ± 1.0^b^	5318.5 ± 52.10^b^	33.1 ± 0.2^b^	2.00 ± 0.1^d^
WPM-640 W	−18.8 ± 0.6^a^	5634.2 ± 138.8^a^	33.6 ± 0.1^a^	2.20 ± 0.1^d^

Means with lowercase superscripts differ significantly (*p* < 0.05).

### 3.2. Zeta potential

The zeta potential of WPM was measured to indicate the electrostatic repulsion between molecules. As shown in Table 1, when the ultrasound power increased from 0 to 640 W/cm^2^, the zeta potential value decreased from−13.80 mV to-18.42 mV. One possible explanation was that ultrasound exposed more polar groups and increased the charged residues on the surface of WPM (15). Zhang et al. (18) also found that ultrasound unfolded the whey protein isolate structure and exposed more negative amino acids. The increase in the absolute value of zeta potential of WPM demonstrated that the electrostatic repulsion between microgel was enhanced, which can inhibit the aggregation of microgels (19). Research on the causes affecting the stability of microgels is gradually underway.

### 3.3. Fluorescence spectroscopy

**Figure 2:**
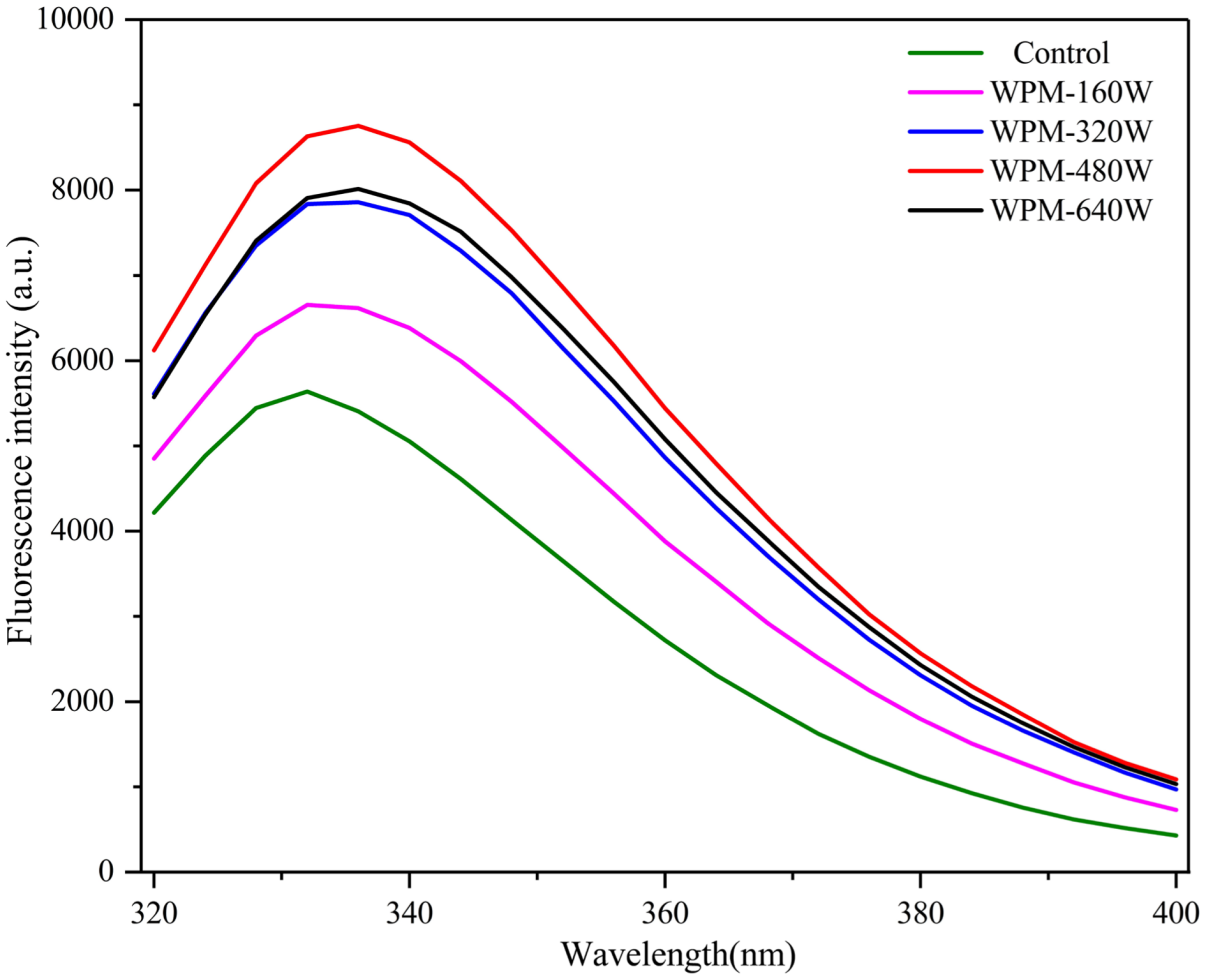
Intrinsic fluorescence of WPM prepared under different ultrasound powers.

Intrinsic fluorescence spectroscopy can characterize the changes in the spatial structure of protein molecules. Therefore, intrinsic fluorescence spectrum analysis was performed on WPM, and the results were illustrated in Figure 2. The maximum emission wavelength (I_max_) of the control and WPM-160 W appeared at 332 nm. However, when the ultrasound power increased to 320, 480, and 640 W/cm^2^, the I_max_ values moved to 336 nm. The reason for the red shift of WPM Imax was that ultrasound disrupted the internal hydrophobic effect, resulting in the unfolding of protein molecules and exposing more chromophores to the surface of protein molecules (10). In addition, the cavitation effect during ultrasound disrupted protein structure, allowing more aromatic amino acids to be exposed, and thus the fluorescence intensity increased significantly after the ultrasound. The slight decrease in fluorescence intensity of WPM-640 W may be attributed to the re-burial of aromatic amino acids by the aggregation of some small fragments.

### 3.4. Surface hydrophobicity

Hydrophobic interactions are essential in maintaining protein stability, and an exogenous fluorescent probe (ANS) is commonly used to assay surface hydrophobicity. The magnitude of surface hydrophobicity can characterize the number of exposed hydrophobic groups. As shown in Table 1, the surface hydrophobicity of WPM increased from 2841.7 to 5634.2 as the ultrasonic power increased to 640 W. During the preparation of WPM, thermal denaturation caused the exposure of hydrophobic regions of whey protein, which was then buried during the formation of aggregates (16). However, after applying ultrasound during heating, whey protein aggregates were disrupted by shear forces, exposing more hydrophobic residues, which was responsible for the increase in surface hydrophobicity of WPM. Furthermore, increasing ultrasound power deepened the unfolding of whey protein and exposed more hydrophobic groups. Therefore, the surface hydrophobicity of samples gradually increased with increasing ultrasound power.

### 3.5. Fourier transform infrared spectroscopy

**Figure 3:**
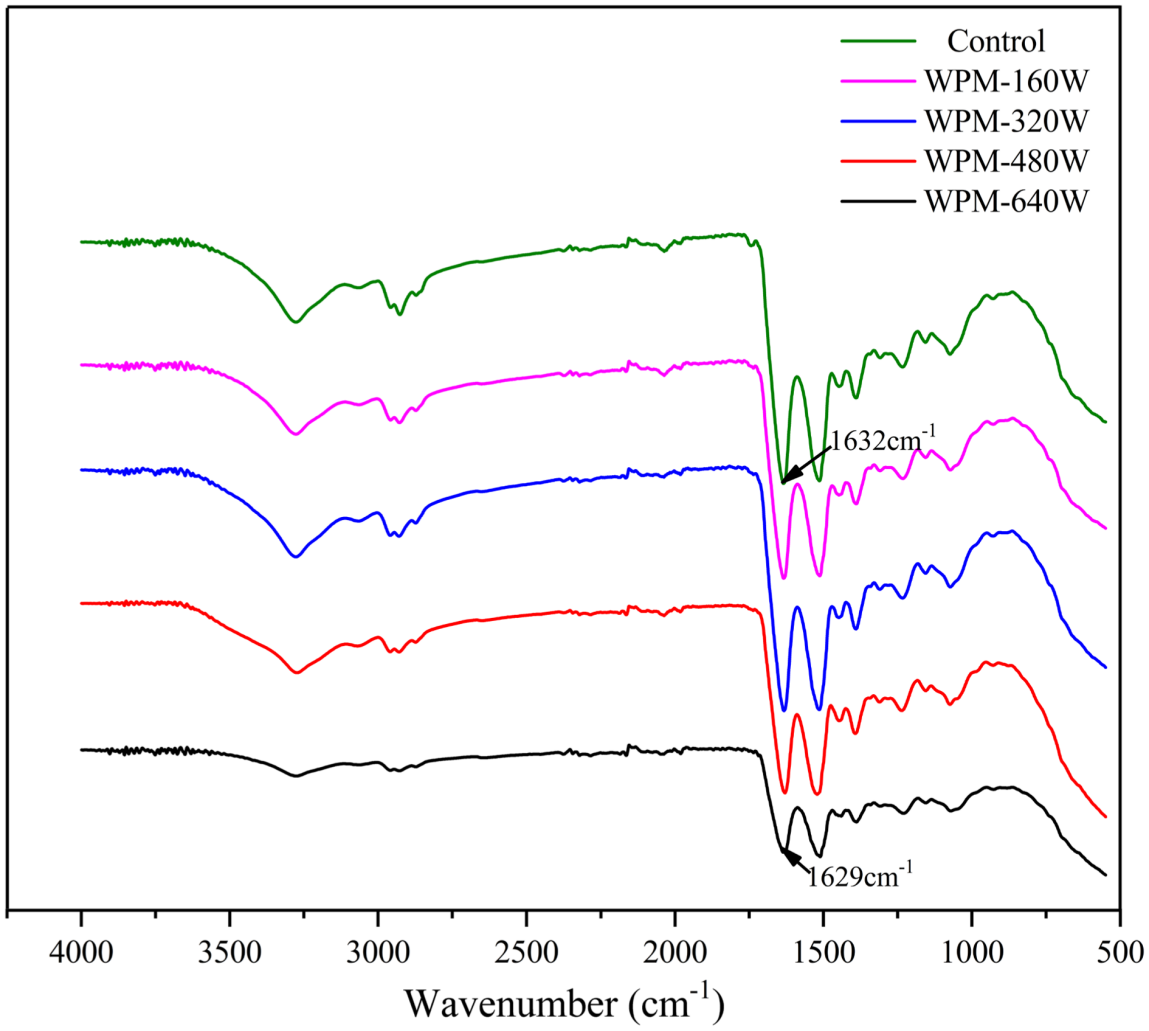
Fourier infrared spectra of WPM under different ultrasound powers.

Fourier infrared spectroscopy is used to analyze the structural changes of WPM by analyzing vibrational band information. The Fourier infrared spectrum of the control and ultrasound-processed WPM is shown in Figure 3. The amide I vibrations of polypeptide chains are very sensitive to changes in secondary structure and are therefore often employed to describe the secondary structure of proteins. The data for the secondary structure content were calculated by fitting to the amide I band and the data were shown in Table 2. The peak of the amide I band of ultrasound-treated WPM changed from 1,629 cm^−1^ to 1,636 cm^−1^ and the change in the β-sheet content in Table 2, demonstrating the increase in β-sheet content (20). Compared with the control, the content of α-helix, β-turn, and the random coil of WPM decreased after the ultrasound. The content of α-helix of WPM-640 W decreased to 13.45%, which proved that ultrasound disrupted the rigid structure of protein molecules to make them more easily unfolded and increase the intermolecular flexibility (21). Ultrasound increased the β-sheet content of WPM, probably attributed to the fact that ultrasound treatment broke some of the hydrogen bonds and converted some of the α-helix structures into β-sheet (22). Yang et al. (23) also demonstrated a significant increment in the β-sheet of wheat proteins following the ultrasound.

**Table 2:** Secondary structure changes of WPM under different ultrasound powers.

Sample	α-helix (%)	β-sheet (%)	β-turn (%)	Random coil (%)
Control	14.59 ± 0.01^a^	42.79 ± 0.03^e^	24.62 ± 0.00^a^	17.99 ± 0.01^a^
WPM-160 W	14.46 ± 0.00^b^	43.31 ± 0.01^d^	24.39 ± 0.01^b^	17.84 ± 0.01^c^
WPM-320 W	13.95 ± 0.00^d^	44.79 ± 0.01^c^	23.35 ± 0.00^c^	17.90 ± 0.00^b^
WPM-480 W	14.01 ± 0.03^c^	45.11 ± 0.01^b^	23.24 ± 0.00^d^	17.59 ± 0.01^d^
WPM-640 W	13.45 ± 0.00^e^	47.56 ± 0.01^a^	22.26 ± 0.01^e^	16.73 ± 0.01^e^

Means with lowercase superscripts differ significantly (*p* < 0.05).

### 3.6. Free sulfhydryl

As shown in Table 1, with increasing the ultrasound power, the sulfhydryl content of WPM increased from 9 to 33.6 μmol/g. This was probably caused by the cavitation effect and shear forces produced from ultrasound, which exposed SH inside the protein molecule or broke the disulfide bond (21). As the ultrasound power increased, the disruption of the protein structure became more apparent. Ultrasound exposed more sulfhydryl inside the whey protein to the surface by disrupting the structure of the protein and reducing the particle size of WPM. The increase in sulfhydryl content with higher ultrasound power was demonstrated during the ultrasound treatment of wheat germ protein (24).

### 3.7. Apparent viscosity

**Figure 4:**
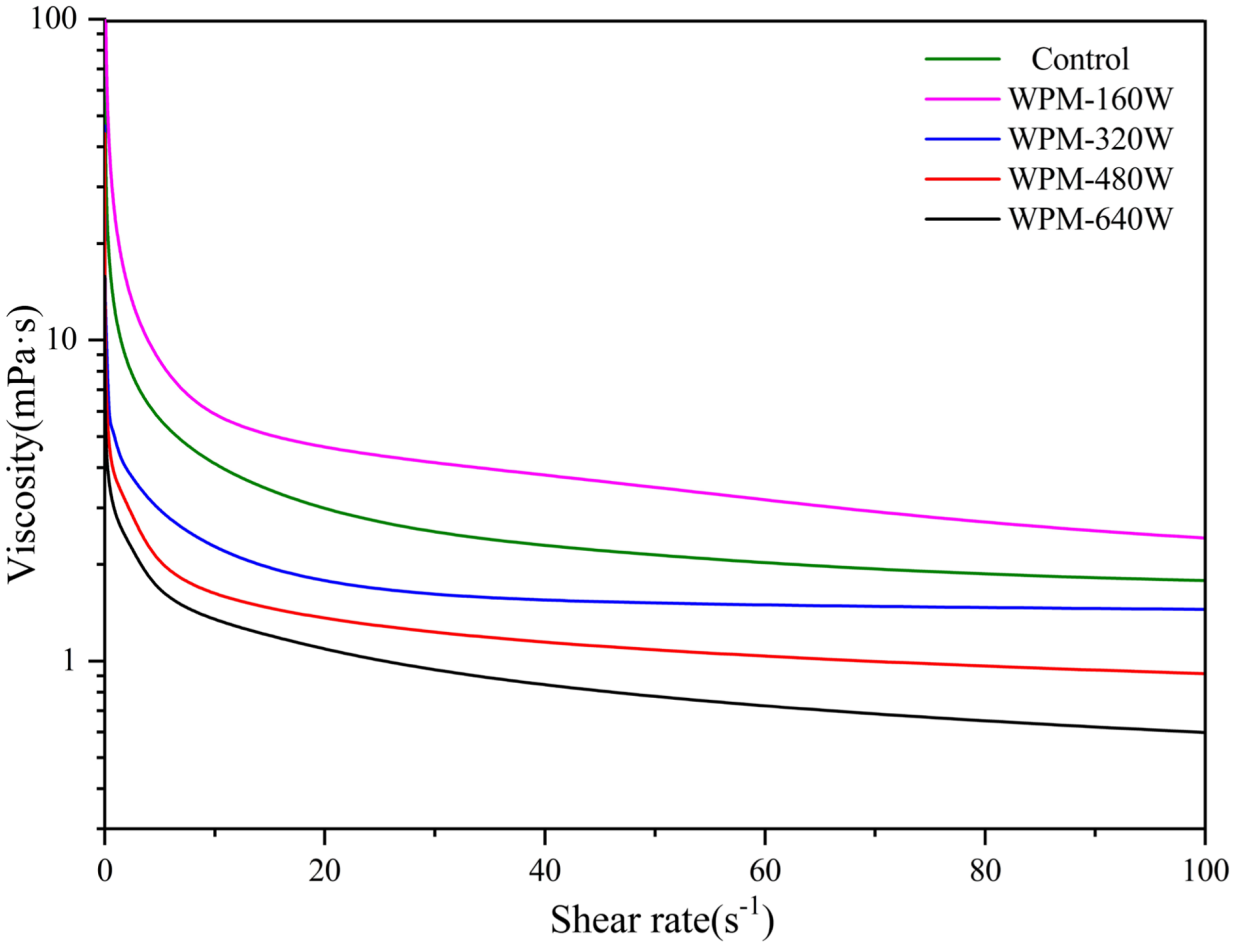
Apparent viscosity of WPM treated with different ultrasonic power.

The extent of cross-linking and interactions between protein molecules affects the apparent viscosity of the solution. The apparent viscosity of WPM is shown in Figure 4. The apparent viscosity of all samples decreased with increasing shear rate, indicating the presence of shear thinning behavior. Compared to the control, only WPM-160 W showed higher apparent viscosity, while the apparent viscosity of other samples decreased with increasing ultrasonic power. The lower ultrasonic power promoted the aggregation of whey protein, which increased the particle size of WPM-160 W and the suspension’s flow resistance, ultimately leading to an increase in the apparent viscosity of the sample (25). However, the apparent viscosity of other samples gradually decreased as the ultrasound power increased. The decrease in apparent viscosity could be related to the reduction in flow resistance and particle size due to the disruption of the interaction of protein molecules by ultrasonic cavitation (26). Zhang et al. (27) reported that ultrasound treatment disrupted the connections between myogenic fibronectin, leading to a decrease in viscosity.

### 3.8. Foam properties

**Figure 5:**
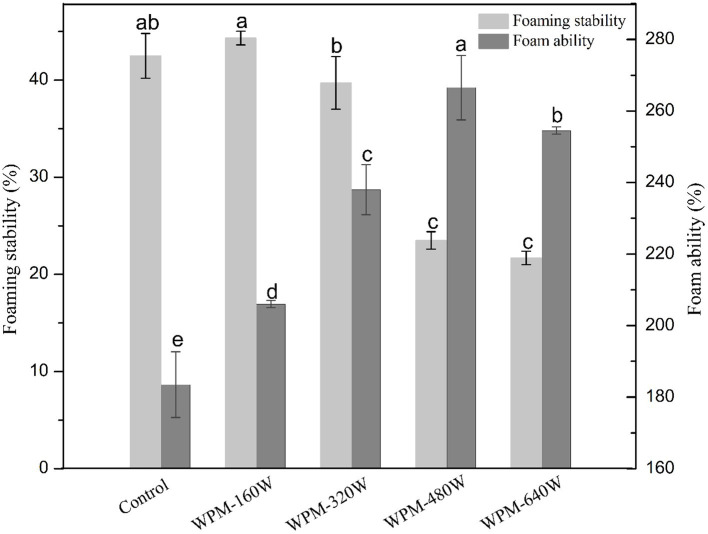
Foaming ability and stability of WPM exposed to various ultrasound powers.

Foamed foods prepared with protein are essential for the human diet. Protein is diffused to the air-water interface by stirring so that the discontinuous air phase is dispersed in the liquid phase to form foam (28). The foam ability and stability of the prepared WPM are shown in Figure 5. Protein foaming is a complex process influenced by numerous factors. The physicochemical properties of the protein, such as solubility, surface hydrophobicity, and particle size, affect the foam properties of the protein (29). Ultrasound treatment dramatically increased the foaming ability of WPM. WPM-480 W showed the highest foam ability, while the control showed the lowest foam ability. This phenomenon may be related to the particle size, flexibility, and apparent viscosity of WPM. Ultrasonic treatment reduced the overall particle size and size distribution range of WPM, which enhanced foaming capacity (30). The reduced content of α-helix led to an increase in protein flexibility and thereby improved the foaming properties. A further reduction in apparent viscosity enhanced the mobility of protein adsorption at the interface and improved the foaming capacity (28). These characteristics of WPM resulted in the shortened timescales for WPM to adsorb to the air-water interface. The foaming ability of WPM-640 W was lower than WPM-480 W. This may be due to the increase in ultrasonic power leading to an increase in temperature, thereby reducing the effectiveness of ultrasound. That resulted in an increase in the average particle size and a decrease in the α-helix structure of WPM-640 W, which affected the protein adsorption at the interface making the foaming ability decrease. While the particle size of WPM-160 W was larger than other samples, foam ability was still higher than the control, which may be due to the increase in surface hydrophobicity. Chang et al. (31) suggested that the exposure of hydrophobic residues could promote egg white protein adsorption to improve foam properties, the finding was similar to the results in this paper.

Regarding foam stability, the amount of foam volume reduction after 10 min can characterize the level of foam stability. Ultrasound is known to modify the structural and physicochemical properties of WPM. These properties affect WPM interactions and adsorption at the air-water interface (32). As shown in Figure 5, compared with the control, the foam stability of WPM-160 W was slightly improved, but other samples showed lower foam stability. The foam stability gradually decreased with increasing ultrasound power. This was attributed to the increased surface hydrophobicity and sulfhydryl content of WPM. The reduction in apparent viscosity made the foam more easily drainage and displayed poor foam stability (18). The increase in surface hydrophobicity made WPM more easily clustered after interfacial desorption, further reducing the foam stability (10). In contrast, the increase in foam stability of WPM-160 W was mainly attributed to its increased viscosity (31), inhibiting foam drainage and thus improving foam stability.

### 3.9. Foam structure

**Figure 6:**
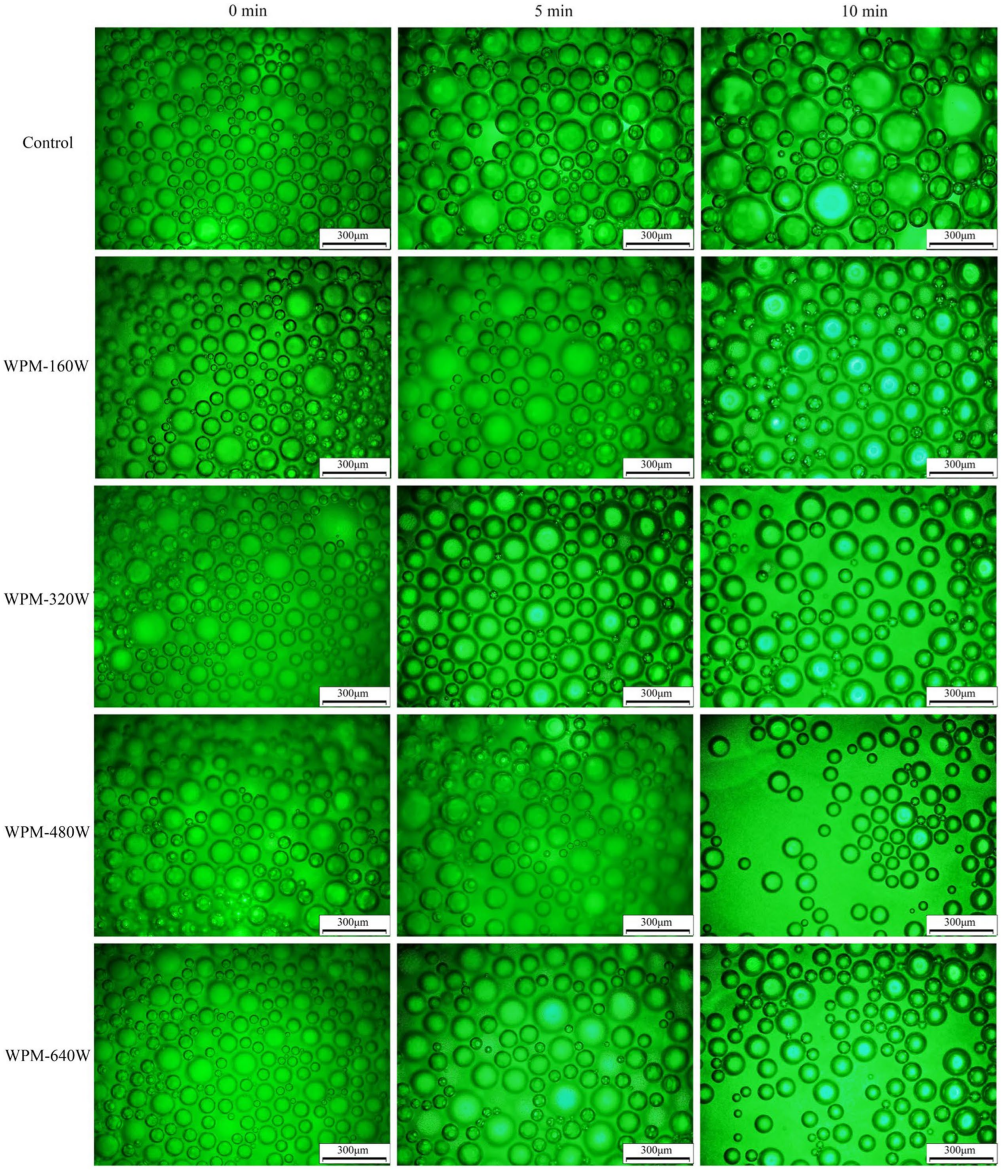
Variation of the morphology of the foams prepared by WPM with time at different ultrasound powers.

**Figure 7:**
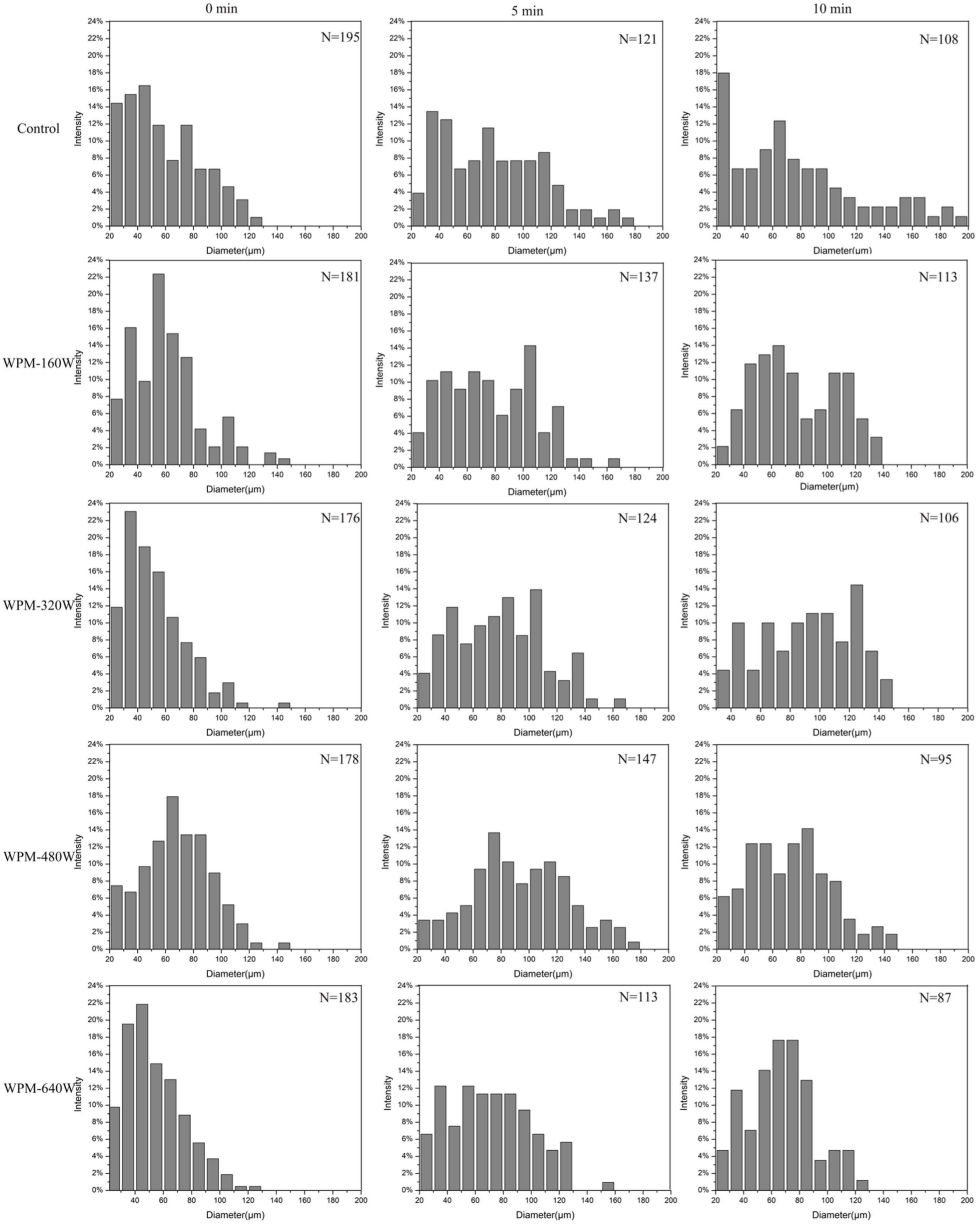
Foam size distribution and the total number of foams at different ultrasound powers. *N* represents the total foam number in the same observation area.

After forming the foam, the diameter of the protein foam gradually increased, and the liquid film became thinner, owing to foam aggregation and gas discharge (31). To intuitively observe the changes in the process of foam dissipating, the foam shape and size under the microscope were observed at different times. The variation of foam morphology for WPM prepared under different ultrasound power is presented in Figure 6. The foam size and quantity were analyzed using Image-Pro. 2.10, and the results were presented in Figure 7. At 0 min, the foams of the control were more uniform in size and numerous than the ultrasound-treated samples. For all samples, at 5 min, the foam number decreased, and the foam size increased slightly. The foam number of the control was observed to decrease from 195 to 121 in the field of view, with a uniform size distribution, and only a small amount of large foams was observed. In contrast, the foam number of WPM-160 W decreased from 181 to 137, the foam size covered the range of 40–100 μm, and no large foam was observed. This may be due to the greater strength of the interface formed during the foaming process of WPM-160 W, which increased the stability of the foam. After 10 min storage, the foam number of WPM decreased due to the diffusion, agglomeration, and disproportionation of foams. The foam number of WPM-160 W was higher than the other samples, which might be due to the fact that WPM-160 W had the largest average particle size and increased surface hydrophobicity and-SH content compared to the control. The foam number of WPM-480 W and WPM-640 W significantly decreased to 95 and 87 after 10 min storage, which may be related to the bursting of larger foam. This probably resulted from the higher hydrophobicity of ultrasound-treated WPM, which tended to cause the foam aggregation to burst more easily. Overall, the generation and change of WPM foam was consistent with the foam capacity and stability results in Section 3.6.

## 4. Conclusion

In this study, we applied ultrasound treatments to the heat preparation process of WPM. Ultrasound disrupted the structure of the WPM, affecting the particle size, and increasing the electrostatic repulsion between molecules, thus affecting the sample’s viscosity. From the perspective of integrated foam formation and stability, the ultrasonic treatment of 320 W was the best choice to obtain the capacity and stability performance. In summary, ultrasound treatment can expand the potential application of WPM in the food industry. The interaction of WPM formation and functional properties needs to be further investigated.

## Data availability statement

The original contributions presented in the study are included in the article/supplementary material, further inquiries can be directed to the corresponding authors.

## Author contributions

ZW: data collection, investigation, experiment, write the first draft, and draw graphs. HZ: assisting with experimentation and software. HT: investigation and data analysis. BY: methodology, analysis, review, and editing. BC: funding acquisition, methodology, review, and supervision. YW: investigation and assisting with experimentation. All authors contributed to the article and approved the submitted version.

## Funding

This work was supported by the University Nursing Program for Young Scholars with Creative Talents in Heilongjiang Province (UNPYSCT-2017157), the Innovation Pilot Project of Integration of Science, Education and Industry of Shandong Province (2022JBZ01-08) and the Fundamental Research Funds of the Department of Education of Heilongjiang Province (135209271).

## Conflict of interest

The authors declare that the research was conducted in the absence of any commercial or financial relationships that could be construed as a potential conflict of interest.

## Publisher’s note

All claims expressed in this article are solely those of the authors and do not necessarily represent those of their affiliated organizations, or those of the publisher, the editors and the reviewers. Any product that may be evaluated in this article, or claim that may be made by its manufacturer, is not guaranteed or endorsed by the publisher.
